# Supplementary material for: Interpretable deep recommender system model for prediction of kinase inhibitor efficacy across cancer cell lines
Source: Sci Rep. 2021 Aug 6;11:15993. doi: 10.1038/s41598-021-94564-z (PMC8346627; doi:10.1038/s41598-021-94564-z)
Supplement: Supplementary file 1 — Supplementary Figures. [file 41598_2021_94564_MOESM1_ESM.pdf]

Interpretable deep recommender system model for prediction of kinase inhibitor efficacy across cancer cell lines
Supplementary Information

Krzysztof Koras[1], Ewa Kizling[1], Dilafruz Juraeva[2], Eike Staub[2], and Ewa Szczurek[1,*]

[1]Faculty of Mathematics, Informatics and Mechanics, University of Warsaw, Warsaw, Poland
[2]Oncology Bioinformatics, Translational Medicine, Merck Healthcare KGaA, Darmstadt, Germany
[*]szczurek@mimuw.edu.pl

1 Supplementary Figures

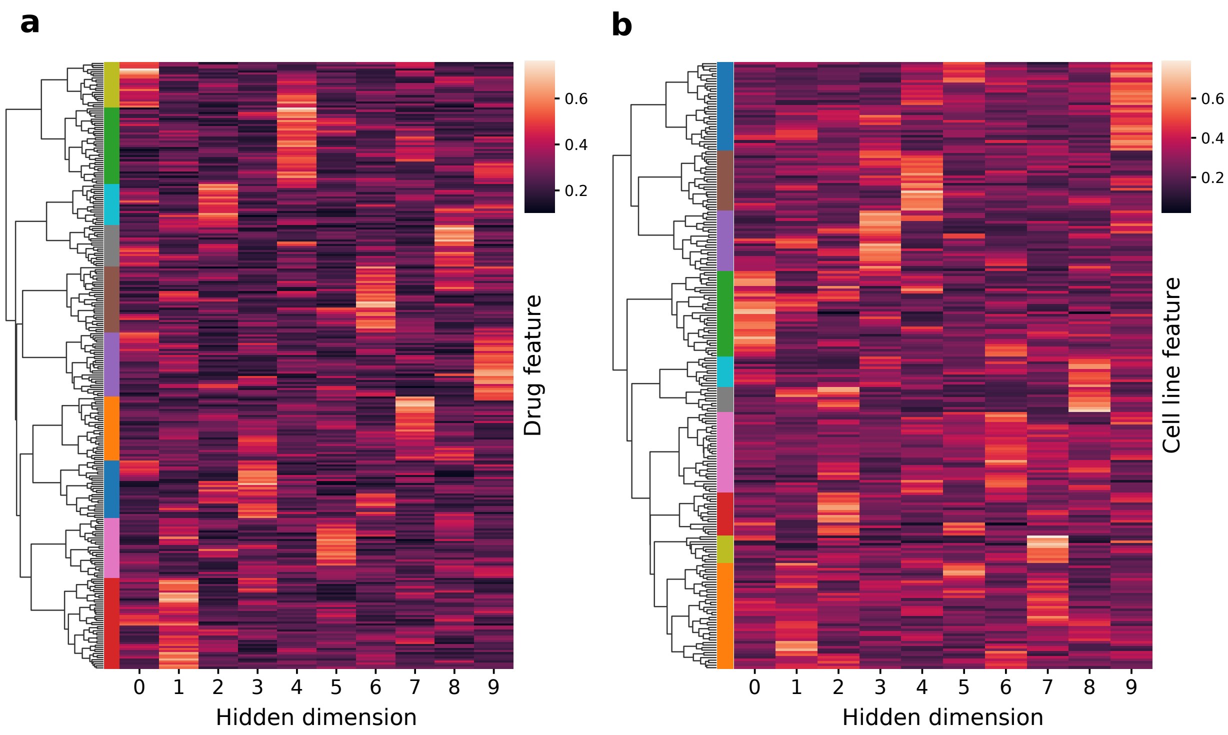

Figure S1. Clustermaps of attribution scores between input features and hidden dimensions for (a) drug autoencoder and (b) cell line autoencoder. Color reflects the importance of a given feature for a given hidden dimension. The vertical color bars next to the dendrograms represent the cluster assignment of groups of features.

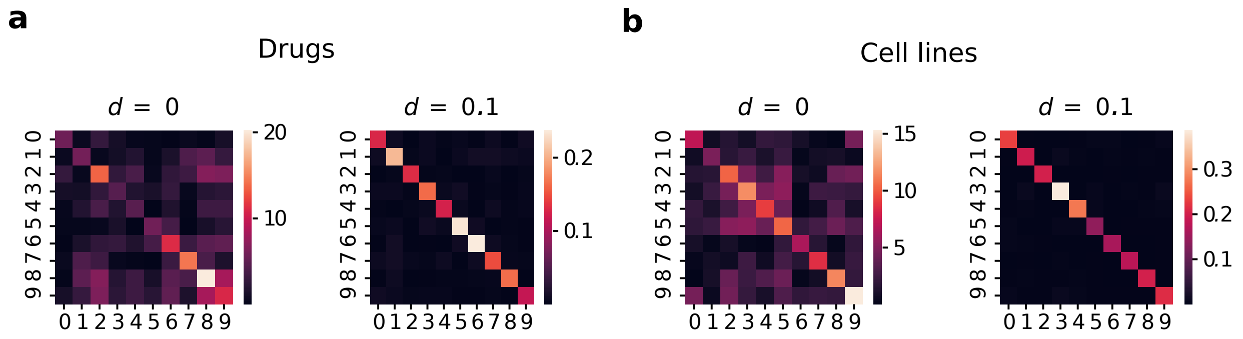

Figure S2. Effect of dependence penalty d shown for (a) drugs and (b) cell lines. First, the drug and cell line data are passed to drug and cell line autoencoders of the DEERS model, respectively, obtaining 10-dimensional hidden representations of all drugs and cell lines used in the analysis. The covariance matrices are calculated across drugs and cell lines in the hidden space. The displayed covariance matrices correspond to the case where DEERS was trained without depencence penalty ($d = 0$), and with dependence penalty term $d = 0.1$.

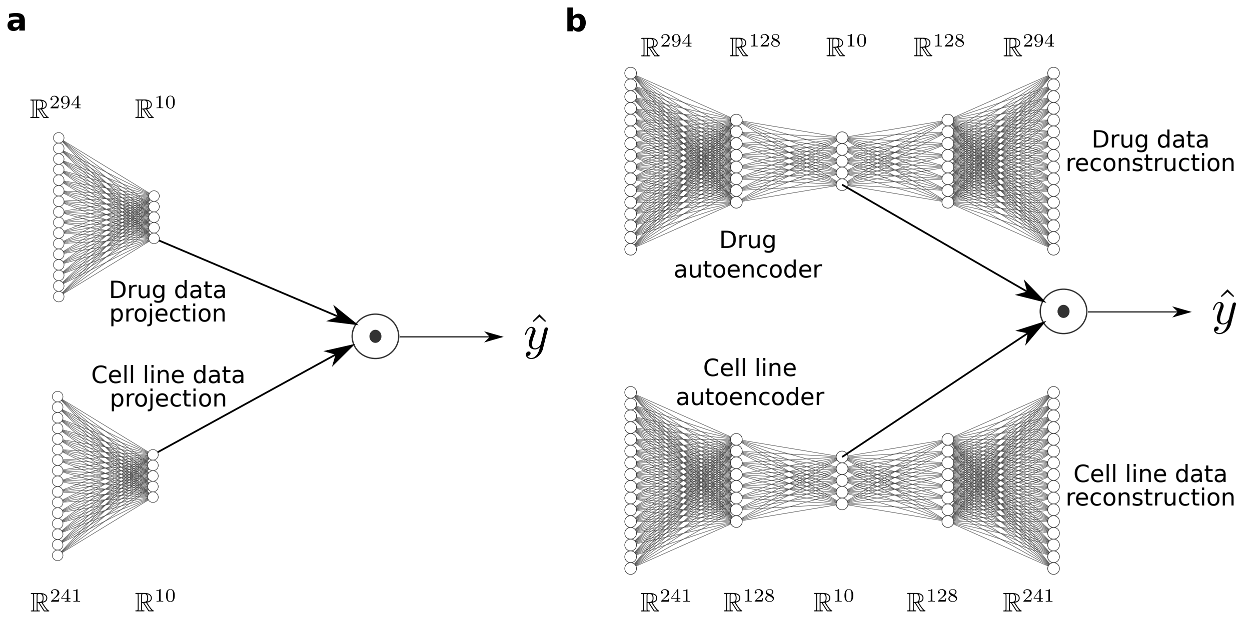

Figure S3. Architecture of the models used for comparison. (a) Linear matrix factorization model (Lin MF). First, drug and cell line data are linearly projected to 10-dimensional hidden representations. Prediction of the response of a cell line to a drug $\hat{y}$ is obtained by applying the dot product to the corresponding hidden representations. **(b)** Non-linear extension of the basic linear model (Autoen MF). The dimensionality reduction is performed via autoencoders with one hidden layer. Prediction of the response of a cell line to a drug is again obtained by taking the dot product of the corresponding, 10-dimensional hidden representations. Drug and cell line data reconstruction errors are also included in the optimization goal.

Supplementary Tables

Table S1. Groups of features corresponding to hidden dimensions of the drug autoencoder in DEERS model. Features are the inhibition strengths of the particular protein kinases.

Table S2. Groups of features corresponding to hidden dimension of the cell line autoencoder in DEERS model. Hidden dimension 5 is omitted due to the lack of clear associated group of features. The “exp” suffix correspond to the gene expresion, “mut” suffix to gene mutation, and “Tissue” prefix reflects the tissue type of the cell line.
